# Elevated neutrophil percentage–to–albumin ratio is associated with postoperative pneumonia in patients with hip fractures

**DOI:** 10.3389/fmed.2026.1796399

**Published:** 2026-05-21

**Authors:** Liuyang Shi, Qionghan He, Zihan Zhang, Jiale Guo, Junjie Xu

**Affiliations:** 1 Department of Orthopedics, The Fourth Affiliated Hospital of Anhui Medical University, Hefei, China; 2 Department of Infectious Diseases, The First Affiliated Hospital of Anhui Medical University, Hefei, China

**Keywords:** composite index, hip fracture, inflammation, nutrition, pneumonia, risk factor

## Abstract

**Background:**

Hip fractures (HF) are common in older adults and are frequently complicated by postoperative pneumonia (POP), which markedly worsens clinical outcomes and increases healthcare burden. Early identification of patients at high risk of POP using simple and readily available indices may facilitate targeted preventive strategies. This study aimed to evaluate the associations between common inflammatory and nutritional indices and the risk of POP in patients with HF.

**Methods:**

Clinical data from patients hospitalized with HF at the Fourth Affiliated Hospital of Anhui Medical University between May 2016 and November 2022 and between March 2023 and May 2025 were retrospectively analyzed. For each composite index, three logistic regression models with progressive adjustment for confounders were constructed to assess associations with POP. Restricted cubic spline (RCS) analyses were applied to indices with significant associations to explore dose–response relationships. Predictive performance was evaluated using receiver operating characteristic (ROC) curves. Model calibration was assessed using the Hosmer-Lemeshow goodness-of-fit test. Subgroup analysis was conducted to examine the stability of the most effective predictive index across different subgroups.

**Results:**

A total of 1,249 patients with HF were included, of whom 122 (9.8%) developed POP. Among ten evaluated indices, the neutrophil percentage–to–albumin ratio (NPAR) showed the strongest predictive performance. The Hosmer–Lemeshow goodness-of-fit test was used to assess model fit, and the results indicated that the model fit well (χ^2^ = 7.11, *df* = 8, *P* = 0.525). NPAR was linearly associated with POP risk, with higher values indicating increased incidence (OR = 1.17, 95% CI 1.11–1.24, *P* < 0.01), and this association remained significant after multivariable adjustment (OR = 1.16, 95% CI 1.09–1.23, *P* < 0.01). In the subgroup analysis, NPAR showed interactions with the three variables: F-type (interaction *P*-value = 0.03), CHD (interaction *P*-value = 0.02), and CIS (interaction *P*-value = 0.01).

**Conclusion:**

NPAR is an independent risk factor for postoperative pneumonia in older patients with hip fractures and demonstrates potential value for early risk stratification.

## 1 Introduction

With the accelerated aging of the population, the quality of life for older adults has become a matter of significant concern (1). Hip fractures (HF) substantially affect geriatric patients' wellbeing, representing a significant public health concern (2, 3). Despite regional declines in hip fracture rates, global incidence was reportedly rising year on year (4). HF incidence is expected to exceed 6.2 million per year by 2050 (5). HF is a prevalent kind of fracture among older patients, posing substantial costs and challenges to patients' families and the healthcare system due to their high disability rate (6). Existing data suggest that China's aggregate hospitalization costs for HF patients during 2012–2016 exceeded US$1 billion (7). Surgical intervention remains the primary treatment approach for HF, offering the advantages of enabling early mobilization, improving prognosis, and reducing mortality rates (8). However, postoperative complications significantly impact the prognosis of HF (9, 10). Approximately one-third of geriatric HF patients reportedly develop complications postoperatively (11).

Postoperative pneumonia (POP) is among the most common complications of HF surgery (12), with its incidence varying across studies, ranging from 3.5% to 15.2% (13–21). The occurrence of POP is not only associated with higher mortality rates and poorer overall prognosis but also markedly prolongs hospital stays (18, 21). Patients experiencing pulmonary infection postoperatively may face a 30-day mortality rate as high as 43% (9). Consequently, the early and accurate prediction of high-risk individuals for POP following HF surgery contributes to optimizing clinical outcomes and improving overall prognosis. In recent years, composite indices derived from laboratory tests have garnered increasing attention for their association with the prognosis of HF. Indices such as PNI (prognostic nutritional index), NLR (neutrophil-to-lymphocyte ratio), NPAR (neutrophil percentage to albumin ratio), and SII (systemic immune inflammation index) were exhibited to correlate with adverse outcomes in HF patients (22–25). A previous study comparing the predictive efficacy of NLR, PLR (platelet-to-lymphocyte ratio), and SII has shown NLR to be the most effective predictor of POP following HF surgery (23). Only three composite indices are included in the above study, yet more and more such indices have been demonstrated to be relevant to disease prognosis.

We aimed to determine the optimal screening index for POP risk stratification in HF surgery patients by methodically assessing the predictive value of 10 popular laboratory composite indices.

## 2 Materials and methods

### 2.1 Data collection

Data on HF patients hospitalized at the Fourth Affiliated Hospital of Anhui Medical University were collected for May 2016–November 2022 and March 2023–May 2025 in this study. Data from December 2022 to February 2023 were not collected due to China's COVID-19 policy adjustments during this period. Our institution's overall approach to hip fractures has remained unchanged over the past decade (during the COVID-19 pandemic, only additional epidemic prevention and control measures were introduced). All patients were treated in accordance with the hospital's standardized clinical pathway for hip fracture surgery. This includes standardized preoperative assessment, surgical procedures, anesthetic management, antibiotic use, and early mobilization following surgery as soon as possible. Relevant medical data concerning the patient was retrieved from the hospital's electronic medical records system. Inclusion criteria: (1) Patients admitted for hip fracture; (2) Patients aged ≥60 years. Exclusion criteria: (1) Patients not undergoing surgical intervention; (2) Preoperative diagnosis of pulmonary infection; (3) Multiple injuries; (4) Missing data exceeding 20%; (5) Presence of acute cardiovascular disease, cerebrovascular disease, cancer, or other conditions seriously compromising patient prognosis; (6) Pathological fractures. The inclusion and exclusion criteria used in this study are identical to those in our previously published article (26).

Pneumonia diagnostic criteria developed by the U.S. Centers for Disease Control and Prevention (CDC) were utilized for POP diagnosis (27): (1) Imaging findings: new pulmonary infiltrative shadows, consolidated lesions, or cavity formation. (2) (a) Fever exceeding 38 °C without any other discernible cause; (b) Leukopenia (white blood cell count < 4 × 10^9^/L) or Leukocytosis (white blood cell count >12 × 10^9^/L); (c) Altered mental status in individuals aged 70 years or older, after ruling out other causes. (3) (a) Increased respiratory secretions, new onset of purulent sputum or altered sputum characteristics, or heightened suctioning requirements; (b) Cough, expectoration, dyspnea, or tachypnea following surgery; (c) Dry rales, wet rales, or abnormal breath sounds; (d) Impaired gas exchange. If a patient concurrently fulfills at least one criterion from (1), at least one from (2), and at least two from (3), and other pulmonary diseases are excluded, a diagnosis of POP can be established. The hospital's medical record system was the source of all relevant data mentioned above. POP is defined as a major outcome occurring between the first postoperative day and discharge.

The variables extracted from our data are primarily categorized into the following three groups: (1) Patient general characteristics; (2) Pre-existing complications; (3) Common preoperative test results within 24 h of admission. Details of all variables are shown in Table 1. Ten commonly used composite indices were calculated based on extracted laboratory tests. The definitions and calculation methods for each indicator are as follows:

(1) Neutrophil-to-lymphocyte ratio (NLR) = neutrophil count (10^9^/L) / lymphocyte count (10^9^/L);
(2) Monocyte-to-lymphocyte ratio (MLR) = monocyte count (10^9^/L) / lymphocyte count (10^9^/L);
(3) Platelet-to-lymphocyte ratio (PLR) = platelet count (10^9^/L) / lymphocyte count (10^9^/L);
(4) Systemic immune inflammation index (SII) = platelet count (10^9^/L) × NLR.
(5) Systemic immune response index (SIRI) = monocyte count (10^9^/L) × NLR.
(6) Aggregate index of systemic inflammation (AISI) = neutrophil count (10^9^/L) × platelet count (10^9^/L) × monocyte count (10^9^/L) / lymphocyte count (10^9^/L).
(7) Hemoglobin, albumin, lymphocyte count, and platelet count (HALP) = hemoglobin (g/L) × albumin (g/L) × lymphocyte count (10^9^/L) / platelet count (10^9^/L);
(8) Prognostic nutritional index (PNI) = albumin (g/L) + 5 × total lymphocyte count (10^9^/L);
(9) Blood urea nitrogen to serum albumin ratio (BAR) = BUN (mg/L) / albumin (g/L);
(10) Neutrophil percentage to albumin ratio (NPAR) = neutrophil percentage (in total WBC count) (%) × 100/albumin (g/dL).

**Table 1:** Basic characteristics of the population included in this study.

Variables	Total (*n* = 1,249)	No-POP (*n* = 1,127)	POP (*n* = 122)	*P*
Sex, *n* (%)				0.31
Female	834 (67)	758 (67)	76 (62)	
Male	415 (33)	369 (33)	46 (38)	
Age, Median (Q1, Q3)	79 (72, 85)	78 (72, 84)	82 (76.25, 86)	< 0.01
F-type, *n* (%)				0.06
FNF	661 (53)	607 (54)	54 (44)	
IFF	588 (47)	520 (46)	68 (56)	
F-side, *n* (%)				0.65
Left	646 (52)	580 (51)	66 (54)	
Right	603 (48)	547 (49)	56 (46)	
F-time[day], Median (Q1, Q3)	1 (1, 2)	1 (1, 2)	1 (1, 2)	0.97
HBP, *n* (%)				0.14
No	612 (49)	544 (48)	68 (56)	
Yes	637 (51)	583 (52)	54 (44)	
CHD, *n* (%)				0.52
No	1,066 (85)	959 (85)	107 (88)	
Yes	183 (15)	168 (15)	15 (12)	
DM, *n* (%)				0.38
No	1,012 (81)	909 (81)	103 (84)	
Yes	237 (19)	218 (19)	19 (16)	
CIS, *n* (%)				0.04
No	912 (73)	833 (74)	79 (65)	
Yes	337 (27)	294 (26)	43 (35)	
CB, *n* (%)				< 0.01
No	1,122 (90)	1,024 (91)	98 (80)	
Yes	127 (10)	103 (9)	24 (20)	
S-time [min], Median (Q1, Q3)	70 (55, 85)	70 (54, 85)	69.5 (57, 90)	0.21
WBC [10^9^/L], Median (Q1, Q3)	7.77 (6.29, 9.51)	7.72 (6.25, 9.43)	8.15 (6.54, 10.55)	0.02
N [10^9^/L], Median (Q1, Q3)	5.95 (4.61, 7.75)	5.89 (4.58, 7.68)	6.47 (4.95, 8.46)	< 0.01
L [10^9^/L], Median (Q1, Q3)	1.01 (0.75, 1.3)	1.01 (0.76, 1.31)	0.95 (0.7, 1.2)	0.24
M [10^9^/L], Median (Q1, Q3)	0.52 (0.4, 0.69)	0.52 (0.4, 0.68)	0.54 (0.39, 0.75)	0.74
HB [g/L], Median (Q1, Q3)	109 (95, 121)	109 (96, 121)	106.5 (87, 119)	0.11
PLT [10^9^/L], Median (Q1, Q3)	149 (115, 190)	148 (115.5, 189)	154.5 (115, 191.75)	0.52
GLU [mmol/L], Median (Q1, Q3)	6.2 (5.4, 7.2)	6.2 (5.4, 7.2)	6 (5.53, 7.5)	0.63
ALT [u/L], Median (Q1, Q3)	16 (12, 22)	16 (12, 22)	15 (11.25, 22.5)	0.19
AST [u/L], Median (Q1, Q3)	22 (18, 27)	22 (18, 27)	22 (18, 29)	0.23
DBIL [umol/L], Median (Q1, Q3)	6 (4.7, 9)	6 (4.7, 9)	7 (4.62, 9)	0.42
IBIL [umol/L], Median (Q1, Q3)	11 (8, 15)	11 (8, 15)	11 (8, 14.15)	0.75
ALB [g/L], Median (Q1, Q3)	37.9 (34.9, 40.5)	38 (35, 40.6)	36 (32.82, 39.48)	< 0.01
GLOB [g/L], Median (Q1, Q3)	27 (24.1, 30.1)	27 (24, 30.1)	27.3 (24.22, 30)	0.78
BUN [mmol/L], Median (Q1, Q3)	6.9 (5.5, 9)	6.9 (5.5, 8.9)	7.3 (5.7, 9.6)	0.31
Cr [umol/L], Median (Q1, Q3)	64 (53, 83)	64 (53, 83)	64 (53.25, 77.85)	0.84
CysC [mg/L], Median (Q1, Q3)	1.07 (0.89, 1.35)	1.07 (0.88, 1.35)	1.1 (0.92, 1.34)	0.57
Ka^+^ [mmol/L], Median (Q1, Q3)	3.8 (3.5, 4.16)	3.8 (3.49, 4.18)	3.8 (3.53, 4.02)	0.66
Na^+^ [mmol/L], Median (Q1, Q3)	140 (138, 142)	140 (138, 142)	140.25 (137.9, 142.2)	0.58
Ca^2+^ [mmol/L], Median (Q1, Q3)	2.15 (2.07, 2.24)	2.15 (2.08, 2.24)	2.12 (2.05, 2.2)	< 0.01
PT [s], Median (Q1, Q3)	12.7 (11.4, 13.6)	12.7 (11.4, 13.6)	12.75 (11.33, 13.7)	0.72
INR, Median (Q1, Q3)	1.01 (0.96, 1.07)	1.01 (0.96, 1.07)	1.02 (0.96, 1.08)	0.44
PTA [%], Median (Q1, Q3)	93.4 (86, 102)	93.4 (86, 102.2)	92.05 (83.55, 100)	0.12
APTT [s], Median (Q1, Q3)	33.5 (28.8, 38.1)	33.5 (28.65, 38.1)	33.65 (29.15, 38.15)	0.37
TT [s], Median (Q1, Q3)	17.1 (16.3, 18)	17.1 (16.3, 18)	17.3 (16.22, 18.4)	0.24
FIB [g/L], Median (Q1, Q3)	3.68 (3.04, 4.59)	3.68 (3.04, 4.55)	3.76 (3.01, 4.89)	0.45
NLR, Median (Q1, Q3)	5.98 (4.15, 8.6)	5.83 (4.1, 8.47)	6.94 (4.72, 9.78)	< 0.01
MLR, Median (Q1, Q3)	0.52 (0.38, 0.72)	0.52 (0.38, 0.71)	0.56 (0.4, 0.73)	0.27
PLR, Median (Q1, Q3)	146.26 (107, 207.68)	145.94 (107.1, 205.7)	156.85 (107.29, 227.64)	0.31
SII, Median (Q1, Q3)	885.13 (573.72, 1,383.7)	865.99 (566.48, 1,369.19)	1,034.15 (698.54, 1,551.86)	< 0.01
SIRI, Median (Q1, Q3)	3.13 (1.94, 5.18)	3.09 (1.92, 5.11)	3.53 (2.31, 5.97)	0.02
AISI, Median (Q1, Q3)	469.73 (262.11, 834.56)	458.84 (259.67, 818.62)	562.7 (312.18, 969.42)	0.02
HALP, Median (Q1, Q3)	26.97 (17.87, 38.88)	27.26 (18.26, 39.28)	23.61 (15.7, 35.92)	0.02
PNI, Median (Q1, Q3)	43.21 (39.71, 46.29)	43.4 (39.91, 46.37)	41.13 (38.03, 44.77)	< 0.01
BAR, Median (Q1, Q3)	5.17 (4.03, 7.04)	5.1 (3.99, 6.92)	5.75 (4.24, 7.77)	0.03
NPAR, Median (Q1, Q3)	20.58 (18.85, 22.72)	20.46 (18.69, 22.5)	22.22 (20.34, 24.9)	< 0.01

F-side, fracture side; F-time, fracture time; F-type, fracture type; S-time, surgery time; HBP, high blood pressure; CHD, coronary heart disease; DM, diabetes mellitus; CIS, cerebral ischemic stroke; CB, chronic bronchitis; WBC, white blood cell count; N, neutrophil count; L, lymphocyte count; M, monocyte count; HB, hemoglobin; PLT, platelet count; GLU, blood glucose; ALT, alanine aminotransferase; AST, aspartate transaminase; DBIL, direct bilirubin; IBIL, indirect bilirubin; ALB, albumin; GLOB, globulin; BUN, blood urea nitrogen; Cr, creatinine; CysC, cystatin C; K^+^, potassium ion; Na^+^, sodium ion; Ca^2+^, calcium ion; PT, prothrombin time; INR, international normalized ratio; PTA, prothrombin time activated; APTT, activated partial thromboplastin time; TT, thrombin time; FIB, fibrinogen; NLR, neutrophil-to-lymphocyte ratio; MLR, monocyte-to-lymphocyte ratio (3); PLR, Platelet-to-lymphocyte ratio; SII, systemic immune inflammation index; SIRI, systemic immune response index; AISI, aggregate index of systemic inflammation; HALP, hemoglobin, albumin, lymphocyte count, and platelet count; PNI, prognostic nutritional index; BAR, blood urea nitrogen to serum albumin ratio; NPAR, neutrophil percentage to albumin ratio.

Approval was granted by the hospital's Ethics Review Committee, and the study was carried out in accordance with the Declaration of Helsinki (ethics number: KYXM-202302-005). As this retrospective study did not involve analysis of identifiable patient information, it was granted exemption from the requirement for informed consent.

### 2.2 Statistical analysis

The handling of missing data constitutes a crucial initial step. First, the “VIM” package is employed to visualize the patterns and structure of missing data, thereby determining the imputation strategy. Subsequently, the “mice” package is utilized for multiple imputation, ensuring the imputation process aligns with the data characteristics. The entire procedure is executed using R software. The Shapiro-Wilk test and quantile-quantile plot were used to examine data normality. Intergroup comparisons were performed using the “CBCgrps” package. Continuous variables meeting the assumption of normality were examined using the *t*-test, while continuous variables with non-normal distributions were analyzed using the Mann-Whitney U-test. Chi-square tests or Fisher's exact tests were applied to categorical variables. Continuous variables meeting normality were expressed as mean ± standard deviation; those with non-normal distribution as median (interquartile range); and categorical variables as percentages (%).

### 2.3 Logistic regression models

To investigate the relationship between POP following HF surgery and the composite index, we initially performed a preliminary univariate logistic regression analysis. Variables and composite indices with *P-values* less than 0.05 were selected for correlation analysis to evaluate the presence of multicollinearity. Correlation coefficients ranged from [−1, 1], with absolute values positively correlated with linear association strength. An absolute value of 0 indicates no linear relationship, a value below 0.4 indicates a weak or weaker correlation, and a value above 0.4 indicates a moderate or stronger correlation. It is generally accepted that when the absolute value of the correlation coefficient is less than 0.4, the linear relationship between variables is weak or even negligible. Therefore, these variables can be retained in the model without concern that they will significantly compromise the model's stability or interpretability. Analysis results were visualized using a heatmap. Variables exhibiting absolute correlation coefficients exceeding 0.4 with composite indices were excluded. Where inter-variable correlations surpassed 0.4, those with larger *P-values* in univariate logistic regression were eliminated.

Three models were constructed for the composite indices screened by univariate logistic regression: Model 1 was unadjusted; Model 2 was adjusted for F-type, CIS, CB, and age; and Model 3 further incorporated adjustments for HB, DM, S-time, and sex based on Model 2. We determine whether the model is overfitted by calculating the events-per-variable (EPV) ratio. It is generally accepted that an EPV of 10 or higher serves as a rule of thumb for effectively reducing the risk of model overfitting and ensuring the reliability of coefficient estimates (28). Additionally, variance inflation factors (VIF) were calculated for all the models for each variable to further verify whether there is multicollinearity. This process further identified composite indices demonstrating stable performance. Odds ratios (OR) with 95% confidence intervals (CI) were employed to report results, with statistical significance defined at *P* < 0.05.

### 2.4 RCS models and ROC curve analysis

Restricted cubic spline (RCS) was employed to explore whether a non-linear relationship exists between composite indices and POP. We constructed receiver operating characteristic curves (ROCs) in order to assess the predictive power of the composite indices. The area under the ROC curve (AUC) reflects a model's predictive performance; the closer the AUC is to 1, the better the predictive performance. Model calibration was assessed using the Hosmer-Lemeshow goodness-of-fit test.

### 2.5 Subgroup analyses

We conducted subgroup analyses to investigate the relationship between the most effective predictive index and the risk of developing POP across various populations. These subgroups included sex, F-side, F-type, HBP, CHD, DM, CIS, and CB.

## 3 Results

### 3.1 Baseline characteristics

**Figure 1:**
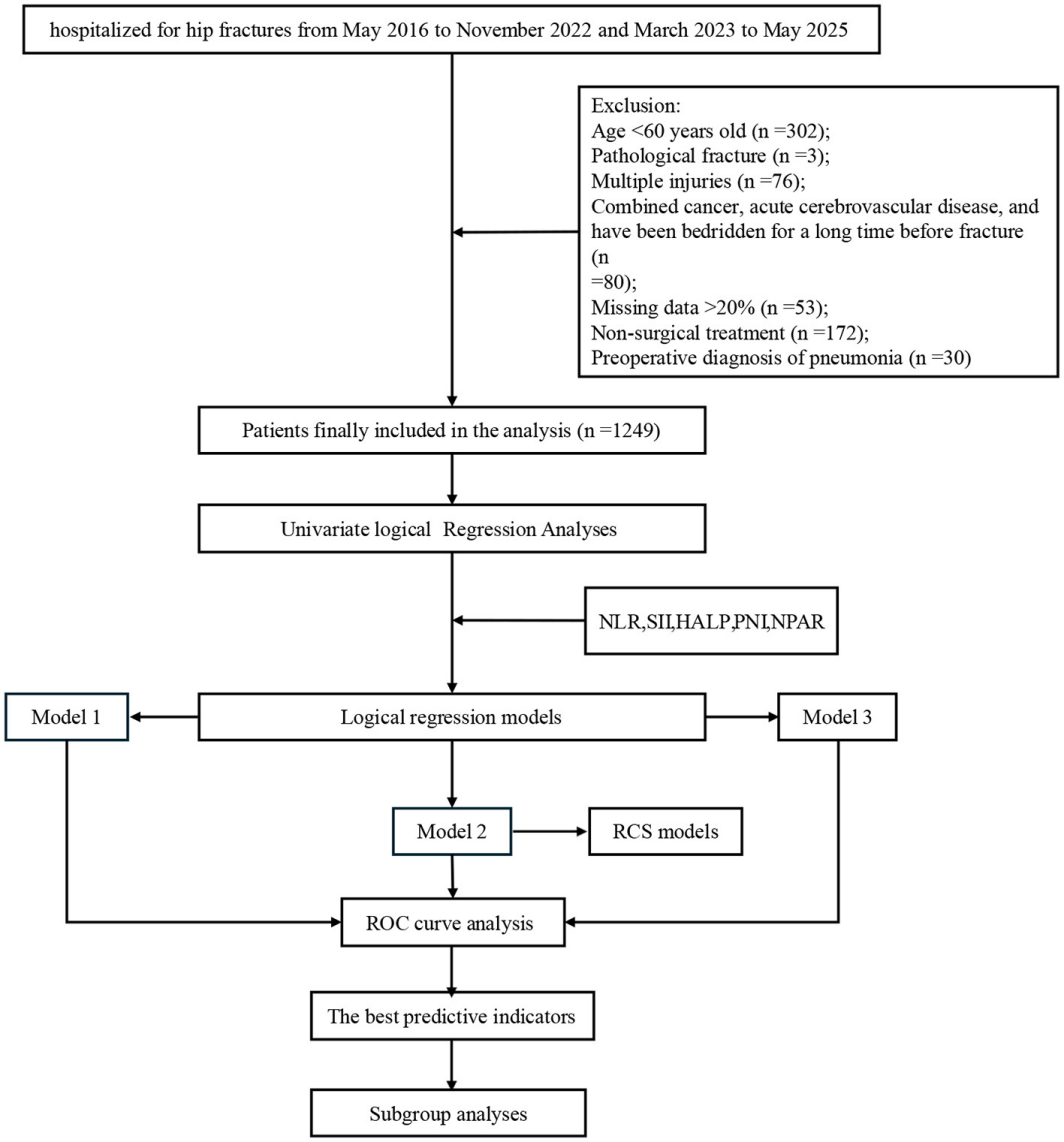
Flowchart of data screening and analysis.

**Figure 2:**
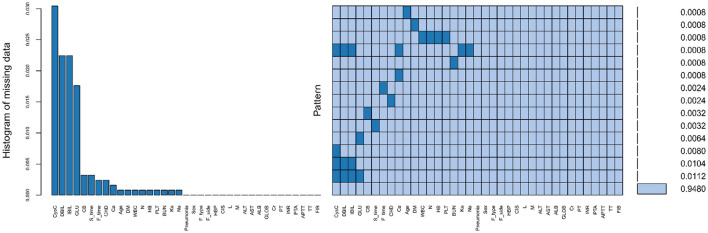
Visualization of missing data. The histogram on the **left** displays the percentage of missing data for each variable, with the vertical axis representing the percentage of missing values for that variable. The heatmap on the **right**, where darker shades indicate missing values, illustrates the distribution of missing data across different variables.

**Figure 3:**
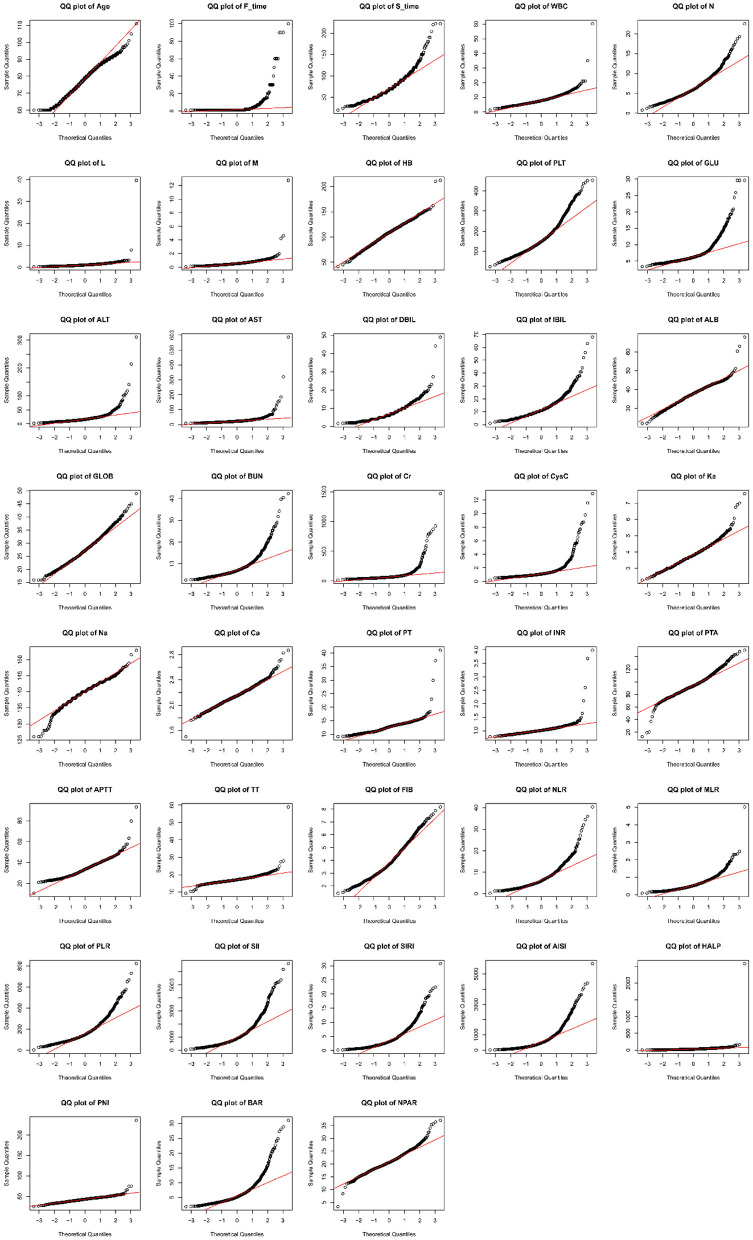
The quantile-quantile plot of all continuous variables. All continuous variables conform to a non-normal distribution. The horizontal axis represents the theoretical quantiles, and the vertical axis represents the sample quantiles. If the data follows a normal distribution, all the points should be roughly distributed along the line of best fit.

Following inclusion and exclusion criteria, a total of 1,249 patients diagnosed with HF were enrolled in the study. The screening and analysis process is illustrated in Figure 1. Across all study variables, missing data rates were below 5% and randomly distributed. Consequently, the results of multiple imputations were reliable (Figure 2). Shapiro-Wilk testing (*P* < 0.05; Figure 3) confirmed the non-normal distribution of all continuous variables, which were expressed as medians (interquartile range). The Mann-Whitney U-test was employed for intergroup comparisons. For HF patients, the median age was 79 years; females represented 834 (67%). Within the sample, 122 (9.8%) developed POP, and among these patients, 62% were female. The samples were analyzed in two groups (POP group and non-POP group). Compared with the non-POP group, the POP group exhibited statistical differences across multiple variable factors. POP patients were older (*P* < 0.001). Regarding comorbidities, concomitant CIS was observed in 35% of POP vs. 26% of non-POP patients; CB occurred in 20% of POP vs. 9% of non-POP patients (*P* < 0.001). In laboratory tests, the POP group had higher indicators of WBC (*P* = 0.021) and neutrophil count (*P* = 0.006) and lower concentrations of ALB (*P* < 0.001) and Ca^2+^ (*P* = 0.007). Regarding the composite indices, the medians of NLR (*P* = 0.005), SII (*P* = 0.008), SIRI (*P* = 0.023), AISI (*P* = 0.02), BAR (*P* = 0.03), and NPAR (P < 0.001) in the POP group significantly increased. However, the medians of HALP (*P* = 0.023) and PIN (*P* < 0.001) showed marked reductions. No significant differences were observed in the remaining variables. The remaining variables showed no statistically significant differences. All baseline characteristics data are detailed in Table 1.

### 3.2 Logistic regression model

**Figure 4:**
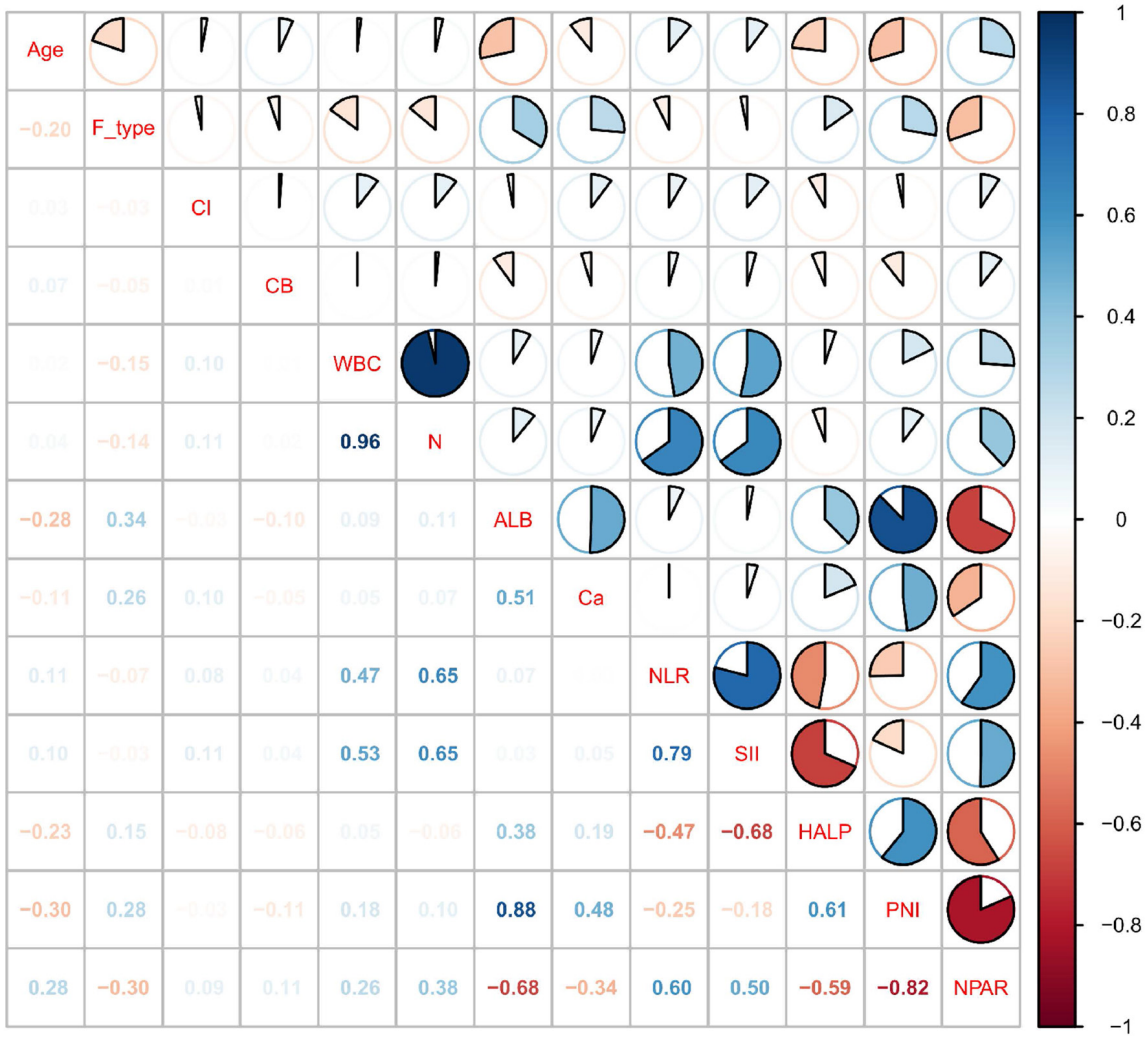
Hotspot plot for correlation analysis of variables.

Using univariate logistic regression analysis, we identified the following variables and composite indices: age, CIS, CB, F-type, WBC, N, ALB, Ca^2+^, NLR, SII, HALP, PIN, and NPAR. All of these variables were *P* < 0.05. Detailed information is shown in Table 2. Incorporating these variables and composite indices into correlation analysis to assess multicollinearity existed, with results presented in Figure 4. Variables exhibiting absolute values of correlation coefficients exceeding 0.4 with composite indices were excluded. Where correlation coefficients between variables surpassed 0.4, the variable with the smaller *P-value* from univariate logistic regression was retained. Variable screening through correlation analysis is only a preliminary step in this study; prior to this process, no assessment of multicollinearity based on VIF was conducted. Ultimately, three logistic regression models were constructed for each composite index: NLR, SII, HALP, PIN, and NPAR. Model 1 made no adjustments; Model 2 adjusted for CB, F-type, CIS, and age; and Model 3 built on Model 2 by further adjusting for HB, DM, S-time, and sex. Model 1 includes only 1 variable, Model 2 includes 5 variables, and Model 3 includes 9 variables. For the 122 POP events, the EPV ratio was 122:1 for Model 1, 24.4:1 for Model 2, and 13.6:1 for Model 3. These ratios exceed the classical threshold (EPV ≥ 10) typically used to obtain reliable logistic regression estimates. There is therefore no risk of overfitting. The VIF for each variable across all models for each composite index was less than 5, further confirming the absence of multicollinearity. Supplementary material 1 provides a detailed breakdown of the composition of each model and the VIF values for all variables. The OR and 95% CI for each composite index within these three model frameworks are presented in Table 3. NLR: Model 1 (OR = 1.04, 95% CI 1.00–1.08, *P* = 0.04), Model 2 (OR = 1.03, 95% CI 0.99–1.07, *P* = 0.23). *P* = 0.17), Model 3 (OR = 1.02, 95% CI 0.98–1.07, *P* = 0.23); SII: Model 1 (OR = 1.00, 95% CI 1.00–1.00, *P* = 0.04), Model 2 (OR = 1.00, 95% CI 1.00–1.00, *P* = 0.16), Model 3 (OR = 1.00, 95% CI 1.00–1.00, *P* = 0.18); HALP: Model 1 (OR = 0.99, 95% CI 0.98–1.00, *P* = 0.04), Model 2 (OR = 0.99, 95% CI 0.98–1.01, *P* = 0.36), Model 3 (OR = 0.99, 95% CI 0.98–1.01, *P* = 0.23); PNI: Model 1 (OR = 0.92, 95% CI 0.88–0.96, *P* < 0.01), Model 2 (OR = 0.94, 95% CI 0.90–0.98, *P* < 0.01), Model 3 (OR = 0.93, 95% CI 0.89–0.97, *P* < 0.01); NPAR: Model 1 (OR = 1.17, 95% CI 1.11–1.24, *P* < 0.01), Model 2 (OR = 1.14, 95% CI 1.08–1.21, *P* < 0.01), Model 3 (OR = 1.16, 95% CI 1.09–1.23, *P* < 0.01). Ultimately, only PNI and NPAR demonstrated consistent results across all three models.

**Table 2:** Univariate regression analyses of risk factors for POP.

Variable	OR (95% CI)	*P*-value
Sex	1.24 (0.84–1.83)	0.27
Age	1.05 (1.02–1.07)	< 0.01
HBP	0.74 (0.51–1.08)	0.12
CHD	0.80 (0.45–1.41)	0.44
DM	0.77 (0.46–1.28)	0.31
CIS	1.54 (1.04–2.29)	0.03
CB	2.43 (1.49–3.98)	< 0.01
F-side	0.90 (0.62–1.31)	0.58
F-time	1.00 (0.97–1.02)	0.79
F-type	1.47 (1.01–2.14)	0.04
S-time	1.00 (1.00–1.01)	0.18
WBC	1.06 (1.01–1.11)	0.03
N	1.11 (1.04–1.19)	< 0.01
L	0.91 (0.63–1.32)	0.61
M	1.02 (0.70–1.50)	0.91
HB	1.00 (0.99–1.01)	0.44
PLT	1.00 (1.00–1.00)	0.45
GLU	1.03 (0.97–1.09)	0.35
ALT	1.00 (0.98–1.01)	0.55
AST	1.00 (1.00–1.01)	0.40
DBIL	1.03 (0.98–1.07)	0.26
IBIL	0.99 (0.97–1.02)	0.71
ALB	0.90 (0.87–0.94)	< 0.01
GLOB	1.01 (0.97–1.05)	0.56
BUN	1.00 (0.96–1.05)	0.92
Cr	1.00 (1.00–1.00)	0.46
CysC	0.97 (0.78–1.19)	0.75
Ka	0.88 (0.63–1.24)	0.47
Na	1.03 (0.97–1.09)	0.36
Ca	0.22 (0.05–0.93)	0.04
PT	0.99 (0.90–1.10)	0.91
INR	0.86 (0.23–3.20)	0.82
PTA	0.99 (0.98–1.01)	0.28
APTT	1.01 (0.98–1.04)	0.44
TT	1.04 (0.96–1.12)	0.37
FIB	1.11 (0.94–1.30)	0.21
NLR	1.04 (1.00–1.08)	0.04
MLR	1.03 (0.59–1.79)	0.92
PLR	1.00 (1.00–1.00)	0.41
SII	1.00 (1.00–1.00)	0.04
SIRI	1.04 (0.99–1.09)	0.13
AISI	1.00 (1.00–1.00)	0.08
HALP	0.99 (0.98–1.00)	0.04
PNI	0.92 (0.88–0.96)	< 0.01
BAR	1.03 (0.98–1.08)	0.21
NPAR	1.17 (1.11–1.24)	< 0.01

**Table 3:** Multivariate regression analyses of risk factors for POP.

Composite index	Model 1		Model 2		Model 3	
	OR (95% CI)	*P*	OR (95% CI)	*P*	OR (95% CI)	*P*
NLR	1.04 (1.00, 1.08)	0.04	1.03 (0.99, 1.07)	0.17	1.02 (0.98, 1.07)	0.23
SII	1.00 (1.00, 1.00)	0.04	1.00 (1.00, 1.00)	0.16	1.00 (1.00, 1.00)	0.18
HALP	0.99 (0.98, 1.00)	0.04	0.99 (0.98, 1.01)	0.36	0.99 (0.98, 1.01)	0.23
PNI	0.92 (0.88, 0.96)	< 0.01	0.94 (0.90, 0.98)	< 0.01	0.93 (0.89, 0.97)	< 0.01
NPAR	1.17 (1.11, 1.24)	< 0.01	1.14 (1.08, 1.21)	< 0.01	1.16 (1.09, 1.23)	< 0.01

### 3.3 Linear relationships and predictive power

**Figure 5:**
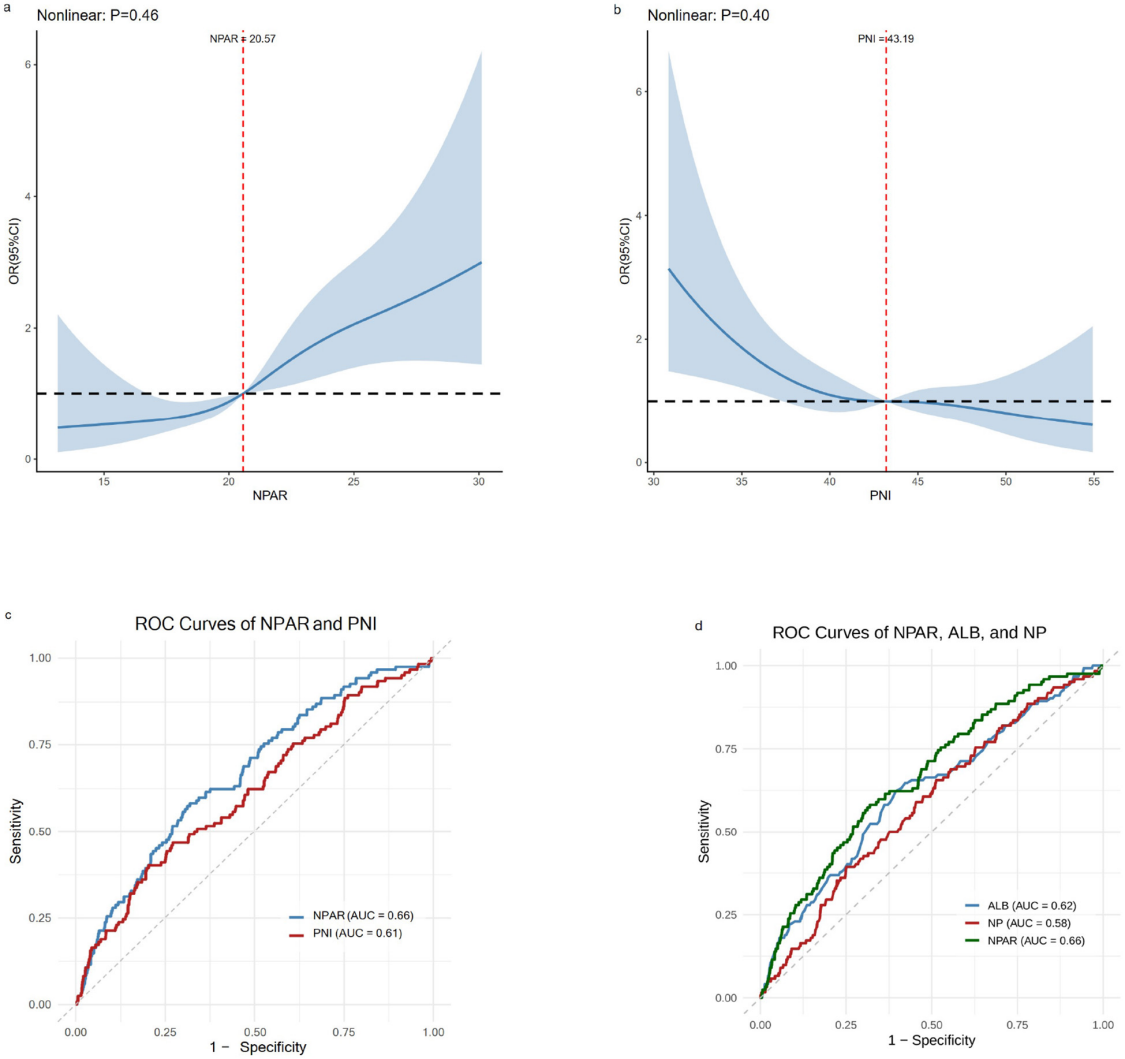
The RCS and ROC of the relevant variables. **(a)** Dose-response relationship between NPAR and POP. **(b)** Dose-response relationship between PNI and POP. **(c)** ROC analysis comparing the predictive ability of NPAR and PIN for POP. **(d)** ROC analysis comparing the predictive ability of NPAR, NP (neutrophil percentage = N / WBC), and ALB for POP.

Due to the excessive number of adjusted variables in Model 3, RCS were plotted based on Model 2 to test the non-linear relationship between composite indices and POP. RCS analysis demonstrated dose-response associations for PNI and NPAR with POP risk. Figure 5a shows no non-linear relationship between NPAR and POP incidence (non-linearity test *P* = 0.46), with a positive correlation observed between the two. Figure 5b demonstrates no non-linear relationship between PNI and POP incidence (non-linearity test *P* = 0.40), with a negative correlation observed. ROC analysis assessed the predictive capability of these composite indices across the entire cohort. Figure 5c displays the ROC curves for NPAR and PNI, with NPAR yielding an AUC value of 0.66 and PNI an AUC value of 0.61. This indicates that NPAR and PNI exhibit certain differences in their predictive capabilities. The Hosmer-Lemeshow test indicated adequate calibration (χ^2^ = 7.11, *df* = 8, *P* = 0.525), with non-significant *P*-values suggesting good agreement between predicted and observed probabilities. Figure 5d illustrates the predictive performance of NPAR, neutrophil percentage, and ALB, respectively. The results show that NPAR (AUC = 0.66) outperformed both the neutrophil percentage alone (AUC = 0.58) and ALB alone (AUC = 0.62).

### 3.4 Subgroup analysis

**Figure 6:**
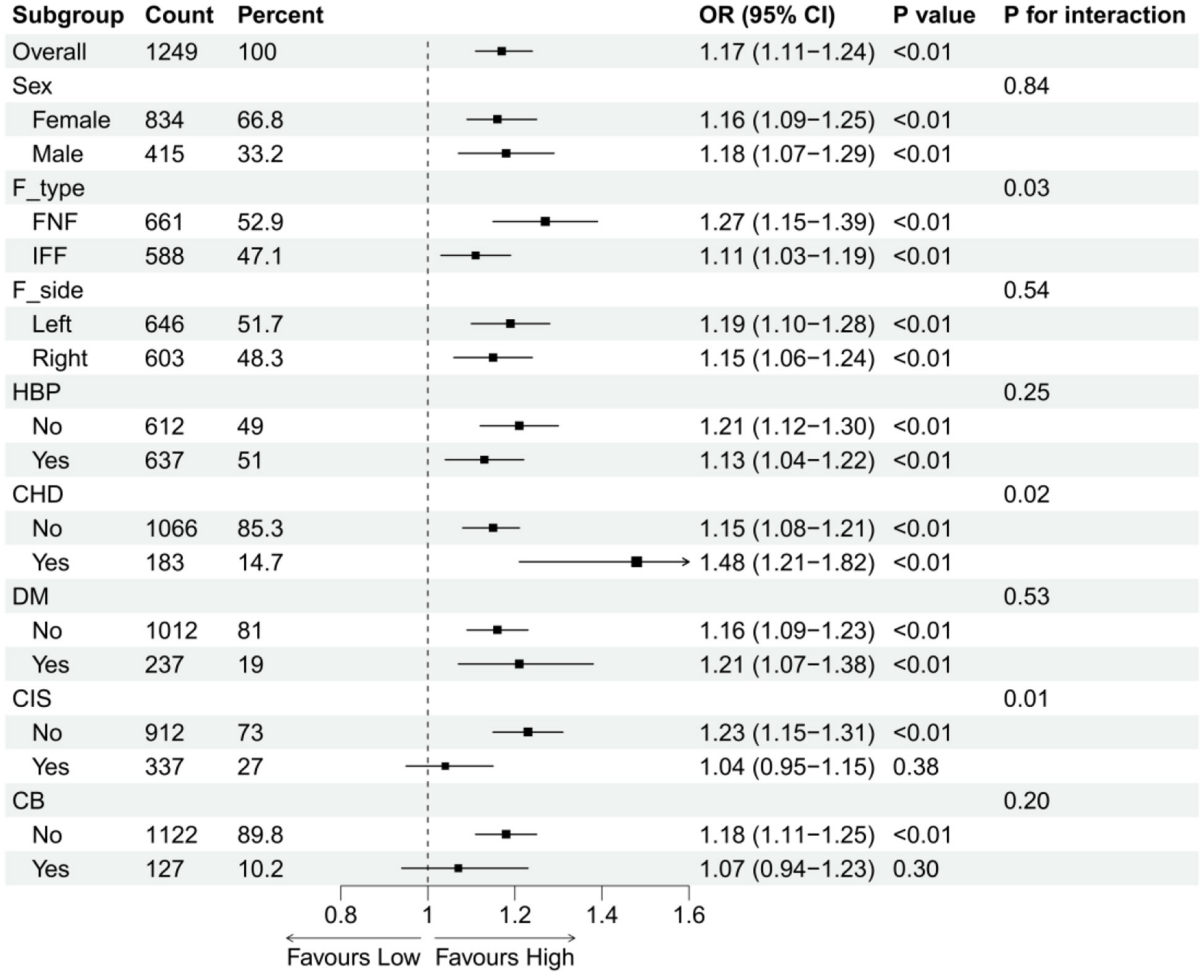
Subgroup analyses of NPAR. Subgroup analyses of older patients with hip fractures were performed to investigate the relationship between the NPAR and POP.

To determine the applicability of NPAR across different populations, we analyzed multiple subgroups. Included variables comprised sex, F-side, F-type, HBP, CHD, DM, CIS, and CB. We observed interactions between NPAR and three variables: F-type (interaction *P*-value = 0.03), CHD (interaction *P*-value = 0.02), and CIS (interaction *P*-value = 0.01). No other factors demonstrated statistically significant interactions. Detailed subgroup characteristics are presented in Figure 6.

## 4 Discussion

This retrospective analysis of 1,249 patients with HF systematically evaluated the predictive value of multiple inflammation and nutrition-related composite indices for POP. Among these composite indices, only PNI and NPAR demonstrated stable and significant correlations with POP. Regarding predictive performance, ROC analysis showed that NPAR outperformed PNI (AUC: 0.66 vs. 0.61) and was superior to neutrophil percentage alone (AUC = 0.58) and albumin alone (AUC = 0.62). PNI, as a composite index of nutritional-immunological status (29), demonstrated a negative correlation (OR < 1) across all three model groups in this study. The mechanism may involve malnutrition suppressing immune cell function, thereby increasing infection risk (30). An observational cohort study by Wang et al., involving 3,351 patients, confirmed that PNI effectively predicted postoperative complications after HF surgery, with higher levels of PNI values being associated with a lower risk of postoperative complications (22). This finding aligned with those of this investigation. Furthermore, a recent meta-analysis also reported similar results (31). Conversely, as a novel index of inflammation and infection, NPAR consistently showed a positive correlation (OR > 1) in the three groups of models, suggesting that it jointly increases the risk of infection through neutrophil-mediated inflammatory responses and decreased albumin levels (reflecting deteriorating nutritional status) (32). This association aligns strongly with the physiological frailty observed in the older adults with HF, where exacerbated inflammation and nutritional depletion synergistically impair respiratory defense mechanisms (33–35). A research result indicates that NPAR levels correlate with postoperative outcomes, with lower values indicating improved prognosis (24). This finding is consistent with our results. RCS further validated that both indices exhibited linear dose-response relationships with POP: PNI showed a linear negative correlation, while NPAR exhibited a linear positive correlation (non-linear test *P* > 0.05). NPAR had a better predictive performance than PNI, potentially because PNI focuses more on nutritional status rather than direct inflammatory response (36), whereas NPAR integrates dual pathways of inflammation and nutrition.

As a straightforward marker reflecting systemic infection and inflammation, elevated levels of NPAR characterize a “high inflammation-low nutrition” state, which has been shown to be a strong predictor of poor prognoses across multiple diseases (37). A retrospective study identified NPAR as an independently associated risk factor for pulmonary infection in cervical spinal cord injury patients (OR = 6.9) (38). Additionally, a US population-based study demonstrated that elevated NPAR correlated with mortality risk and exhibited superior predictive performance for 5-year all-cause mortality vs. other hematological inflammatory biomarkers. (39). Moreover, in a cross-sectional study, Su et al. confirmed NPAR serves as a dual-purpose composite index for both breast cancer incidence risk assessment and prognostic evaluation: the NPAR demonstrated significant positive correlations with both breast cancer incidence risk and disease-specific mortality (40). For the HF, only one multicenter study demonstrated NPAR's independent predictive value for 1-year postoperative all-cause mortality, with elevated levels frequently indicating increased mortality risk (24). The application of NPAR to predict POP in patients with HF has not yet been examined in any previous research. This study focuses for the first time on the prognosis of POP in patients with HF and finds that NPAR is significantly elevated in the population with POP.

NPAR has been shown to be useful in predicting the onset of POP among patients who suffered HF, according to our research, but its precise mechanism of action remains unclear. NPAR incorporates neutrophil percentage and albumin levels to reflect a patient's nutritional condition, infection status, and inflammatory state (41). All these factors potentially contribute to POP risk following HF procedures. An elevated percentage of neutrophils or reduced albumin levels will both cause an increase in the NPAR value. Neutrophils, as a key component of the NPAR, are the predominant WBC type in the peripheral blood and play a vital role in the host's immune defense against microbial invasion (42). Neutrophils, serving as phagocytic cells, represent the first responders to infectious sites, where they release reactive oxygen species (ROS) and other bioactive substances during the mediation of inflammatory responses to combat external pathogens, thereby forming the body's primary line of immune defense (43). Following injury or infection, neutrophils can rapidly migrate from the circulation to the site of tissue damage or infection under the influence of selectin, chemokines, and integrins (44). In addition, during immune responses, neutrophils undergo rapid proliferation and differentiation under the influence of granulocyte colony-stimulating factor (G-CSF), with subsequent bone marrow release into peripheral blood (45). This leads to an elevated percentage of peripheral blood neutrophils in laboratory examinations, accompanied by an increase in the NPAR value. Numerous studies have now confirmed that neutrophils serve as a significant indicator of infection (46). However, research has demonstrated that neutrophils can release a variety of chemicals that harm their own tissues (47, 48). In a rat experimental model, HF and surgery can trigger systemic aseptic inflammation, leading to elevated neutrophil levels, causing pulmonary injury, and increasing the risk of pulmonary infection (49). As the second component of NPAR, albumin is the primary protein in blood, reflecting the body's nutritional status (50). Its roles encompass buffering, extracellular antioxidation, immunomodulation, detoxification, and plasma protein transport (50, 51). Hypoalbuminemia reflects malnutrition and systemic inflammation, constituting a recognized predictor of poor prognosis, being associated with morbidity and mortality (52, 53). Hypoalbuminemia correlates with the occurrence and severity of infectious diseases (54). Insufficient protein intake among older adults leads to lower albumin levels at hospital admission; this form of malnutrition, which is a form of qualitative rather than quantitative malnutrition, is associated with frailty (55, 56). According to a systematic review, albumin is a key indicator of the severity of the disease, and critically ill COVID-19 patients have much lower albumin levels at admission than non-critical groups (57). Chung et al. (58) confirmed a positive correlation between albumin levels and the severity of 30-day postoperative complications following HF, with pulmonary and infection-related sequelae being most pronounced. Research indicates that hypoalbuminemia impairs innate and adaptive immune responses, increasing the risk of primary and secondary infections (54). Gau et al. (59) demonstrated that a 1 g/dL serum albumin reduction corresponded to a 1.89-fold elevation in community-acquired pneumonia (CAP) risk among older adults. Tian et al. (60) reported that preoperative hypoalbuminemia independently predicted POP in their cohort of 426 older adults undergoing HF surgery, conferring a 6.18-fold risk elevation. A recent study discovered a bidirectional association between POP in the older adults with HF and hypoalbuminemia: preoperative hypoalbuminemia elevates the risk of POP, whilst POP itself leads to further declines in albumin levels, creating a vicious cycle that adversely impacts patient prognosis (61). In the present study, our findings similarly suggest an association between albumin levels and POP (OR = 0.9).

Subgroup analyses further validated the stability of the overall association between NPAR and the probability of hip fracture (POP), although significant interactions were observed in the F-type, presence of CHD, and CIS subgroups. Overall, elevated NPAR was significantly associated with an increased risk of POP, and the direction of the effect remained consistent across most subgroups. Although interactions were observed in the F-type and CHD strata, the positive association between NPAR and POP remained unchanged, with no evidence of heterogeneity. However, this positive association was more pronounced in patients with FNF and those with concomitant CHD. It is worth noting that the positive association between NPAR and POP was no longer significant in patients with concomitant CIS, suggesting marked heterogeneity. These findings suggest that the relationship between inflammation-nutrition-related indicators and POP is not entirely homogeneous but may be influenced by specific comorbid conditions, particularly neurological disorders. In other words, whilst high NPAR may serve as an important indicator of increased POP risk in the majority of hip fracture patients, this association may be attenuated in patients with concomitant cerebral infarction due to factors such as neurological dysfunction, impaired swallowing function, increased risk of aspiration, and reduced airway protection capacity. Furthermore, the observed interactions may also be attributable to insufficient sample size; in this study, there were 183 patients with CHD and 337 patients with CIS; specifically, in the POP group, there were only 15 patients with CHD and 43 patients with CIS, representing a relatively small sample size. The disparity in sample size between the two groups may have led to differences in statistical power, potentially preventing the detection of a genuinely existing association. Although the interaction test was significant, the clinical significance of the effect modification may be limited.

This study introduces NPAR into the predictive analysis of POP after HF for the first time. NPAR reflects both nutritional and inflammatory states, with its levels jointly regulated by neutrophil and albumin concentrations. HF activates inflammatory responses leading to elevated neutrophil counts, while older HF patients frequently present with concomitant malnutrition (24). Therefore, compared to increases in neutrophil levels or decreases in albumin levels, NPAR amplifies the predictive effects of both, establishing a stronger association with POP following HF. This enables more accurate prediction of the risk of POP occurrence. Overall, NPAR serves as a favorable index for predicting POP in older HF patients. By integrating both inflammatory and nutritional indicators, it constitutes a more reliable composite index. We therefore recommend that appropriate clinical measures be taken for patients with elevated NPAR. These measures may include post-operative chest X-rays, sputum cultures, and more rigorous management of the lungs and respiratory tract.

However, this study also has several limitations. First, it suffers from selection bias, being a retrospective study. Secondly, although the sample size was large, the number of POP events (*n* = 122) remains relatively limited for a multifactorial model with multiple variables. This may restrict the model's capacity for more complex interaction analyses and indicates that future validation studies with larger samples are required. Thirdly, although this study made every methodological effort to account for the influence of known confounding factors, because it is a retrospective study, variables such as lung function, COPD, and smoking—which, as the reviewers noted, are important factors in the development of postoperative pneumonia—were not recorded at the time of admission. For example, functional status measures (e.g., activities of daily living), ASA physical status classification, types of antibiotics, postoperative ventilatory support, and oral hygiene. Fourthly, in the subgroup analysis of this study, interactions were observed within the three subgroups of F-type, whether combined with CHD and CIS, suggesting that there are differences in the correlation between NPAR and POP within different subgroups. Given the small number of patients with concomitant CHD or CIS in this study, further evaluation of NPAR's predictive performance within these subgroups was not pursued. Future studies with larger sample sizes are required to investigate these distinct patient groups separately. Fifthly, the AUC value for NPAR is 0.66, which is considered moderate and is insufficient to accurately predict individual risk. This indicator should only be used as a simple, rapid method for screening for the risk of postoperative pneumonia; patients will still need to undergo further assessment of their risk of postoperative pneumonia. Sixth, this study is subject to temporal bias. Over the past decade, surgeons' technical proficiency has improved, anesthesiologists have gained more clinical experience, the types of antibiotics available have changed, and surgical implants have been improved—all of which are factors that cannot be overlooked. Finally, the retrospective and single-center nature of the study limits generalizability, and the absence of external validation restricts the immediate clinical applicability of the proposed model. Consequently, future research should incorporate multicenter prospective studies for external validation.

## 5 Conclusion

NPAR, a novel and readily accessible composite index, is closely associated with POP in HF patients and exhibits a certain degree of predictive capability.

## Data Availability

The original contributions presented in the study are included in the article/Supplementary material, further inquiries can be directed to the corresponding authors.
