# Supplementary material for: Elevated neutrophil percentage–to–albumin ratio is associated with postoperative pneumonia in patients with hip fractures
Source: Front Med (Lausanne). 2026 May 21;13:1796399. doi: 10.3389/fmed.2026.1796399 (PMC13233207; doi:10.3389/fmed.2026.1796399)
Supplement: Supplementary file 1 [file Table_1.docx]

**Supplementary Material 1:** The composition of each model within the various composite indices and the VIF values for all variables (to calculate the variance inflation factor, there must be at least two variables).

Supplementary Table 1. The composition of each model of NPAR and the VIF values of all variables.

Supplementary Table 1-1. Model 1: No variables adjusted.

| Variable | OR | Lower-CI | Upper-CI | P-value |
| --- | --- | --- | --- | --- |
| NPAR | 1.17 | 1.11 | 1.24 | <0.01 |

Supplementary Table 1-2. Model 2: Adjusted for age, F-type, CIS, and CB.

| Variable | OR | Lower-CI | Upper-CI | P-value | VIF |
| --- | --- | --- | --- | --- | --- |
| Age | 1.03 | 1.00 | 1.06 | 0.02 | 1.08 |
| F-type | 1.00 | 0.67 | 1.50 | 1.00 | 1.11 |
| CIS | 1.46 | 0.98 | 2.19 | 0.07 | 1.00 |
| CB | 2.08 | 1.26 | 3.45 | <0.01 | 1.01 |
| NPAR | 1.14 | 1.08 | 1.21 | <0.01 | 1.14 |

Supplementary Table 1-3. Model 3: Adjusted for age, F-type, CIS, CB, sex, DM, S-time, and HB.

| Variable | OR | Lower-CI | Upper-CI | P-value | VIF |
| --- | --- | --- | --- | --- | --- |
| Age | 1.03 | 1.01 | 1.06 | 0.01 | 1.16 |
| F-type | 1.09 | 0.69 | 1.72 | 0.72 | 1.40 |
| CIS | 1.51 | 1.00 | 2.26 | 0.05 | 1.02 |
| CB | 2.02 | 1.21 | 3.37 | 0.01 | 1.04 |
| Sex | 1.05 | 0.69 | 1.60 | 0.81 | 1.10 |
| DM | 0.84 | 0.49 | 1.43 | 0.51 | 1.06 |
| S-time | 1.00 | 1.00 | 1.01 | 0.31 | 1.15 |
| HB | 1.01 | 1.00 | 1.02 | 0.07 | 1.43 |
| NPAR | 1.16 | 1.09 | 1.23 | <0.01 | 1.25 |

Supplementary Table 2. The composition of each model of PNI and the VIF values of all variables.

Supplementary Table 2-1. Model 1: No variables adjusted.

| Variable | OR | Lower-CI | Upper-CI | P-value |
| --- | --- | --- | --- | --- |
| PNI | 0.92 | 0.88 | 0.96 | <0.01 |

Supplementary Table 2-2. Model 2: Adjusted for age, F-type, CIS, and CB.

| Variable | OR | Lower-CI | Upper-CI | P-value | VIF |
| --- | --- | --- | --- | --- | --- |
| Age | 1.03 | 1.01 | 1.06 | 0.01 | 1.08 |
| F-type | 1.10 | 0.74 | 1.64 | 0.64 | 1.09 |
| CIS | 1.52 | 1.02 | 2.26 | 0.04 | 1.00 |
| CB | 2.10 | 1.27 | 3.47 | <0.01 | 1.01 |
| PNI | 0.94 | 0.90 | 0.98 | <0.01 | 1.14 |

Supplementary Table 2-3. Model 3: Adjusted for age, F-type, CIS, CB, sex, DM, S-time, and HB.

| Variable | OR | Lower-CI | Upper-CI | P-value | VIF |
| --- | --- | --- | --- | --- | --- |
| Age | 1.04 | 1.01 | 1.06 | <0.01 | 1.16 |
| F-type | 1.17 | 0.74 | 1.83 | 0.50 | 1.39 |
| CIS | 1.56 | 1.04 | 2.35 | 0.03 | 1.02 |
| CB | 2.05 | 1.23 | 3.41 | <0.01 | 1.04 |
| Sex | 1.01 | 0.66 | 1.54 | 0.96 | 1.13 |
| DM | 0.86 | 0.51 | 1.47 | 0.59 | 1.05 |
| S-time | 1.00 | 1.00 | 1.01 | 0.19 | 1.15 |
| HB | 1.01 | 1.00 | 1.02 | 0.08 | 1.49 |
| PNI | 0.93 | 0.89 | 0.97 | <0.01 | 1.32 |

Supplementary Table 3. The composition of each model of HALP and the VIF values of all variables.

Supplementary Table 3-1. Model 1: No variables adjusted.

| Variable | OR | Lower-CI | Upper-CI | P-value |
| --- | --- | --- | --- | --- |
| HALP | 0.99 | 0.98 | 1.00 | 0.04 |

Supplementary Table 3-2. Model 2: Adjusted for age, F-type, CIS, and CB.

| Variable | OR | Lower-CI | Upper-CI | P-value | VIF |
| --- | --- | --- | --- | --- | --- |
| Age | 1.04 | 1.01 | 1.07 | <0.01 | 1.07 |
| F-typeIFF | 1.23 | 0.84 | 1.82 | 0.29 | 1.04 |
| CIS | 1.50 | 1.00 | 2.24 | 0.05 | 1.01 |
| CB | 2.23 | 1.36 | 3.67 | <0.01 | 1.01 |
| HALP | 0.99 | 0.98 | 1.01 | 0.36 | 1.06 |

Supplementary Table 3-3. Model 3: Adjusted for age, F-type, CIS, CB, sex, DM, S-time, and HB.

| Variable | OR | Lower-CI | Upper-CI | P-value | VIF |
| --- | --- | --- | --- | --- | --- |
| Age | 1.04 | 1.02 | 1.07 | <0.01 | 1.14 |
| F-type | 1.23 | 0.78 | 1.93 | 0.37 | 1.41 |
| CIS | 1.51 | 1.01 | 2.27 | 0.04 | 1.02 |
| CB | 2.15 | 1.30 | 3.57 | <0.01 | 1.04 |
| Sex | 1.14 | 0.75 | 1.71 | 0.55 | 1.10 |
| DM | 0.85 | 0.50 | 1.45 | 0.56 | 1.05 |
| S-time | 1.01 | 1.00 | 1.01 | 0.17 | 1.15 |
| HB | 1.01 | 0.99 | 1.02 | 0.31 | 1.52 |
| HALP | 0.99 | 0.98 | 1.01 | 0.23 | 1.21 |

Supplementary Table 4. The composition of each model of SII and the VIF values of all variables.

Supplementary Table 4-1. Model 1: No variables adjusted.

| Variable | OR | Lower-CI | Upper-CI | P-value |
| --- | --- | --- | --- | --- |
| SII | 1.00 | 1.00 | 1.00 | 0.04 |

Supplementary Table 4-2. Model 2: Adjusted for age, F-type, CIS, and CB.

| Variable | OR | Lower-CI | Upper-CI | P-value | VIF |
| --- | --- | --- | --- | --- | --- |
| Age | 1.04 | 1.02 | 1.07 | <0.01 | 1.03 |
| F-type | 1.26 | 0.85 | 1.85 | 0.24 | 1.03 |
| CIS | 1.48 | 0.99 | 2.22 | 0.05 | 1.01 |
| CB | 2.24 | 1.36 | 3.68 | <0.01 | 1.01 |
| SII | 1.00 | 1.00 | 1.00 | 0.16 | 1.01 |

Supplementary Table 4-3. Model 3: Adjusted for age, F-type, CIS, CB, sex, DM, S-time, and HB.

| Variable | OR | Lower-CI | Upper-CI | P-value | VIF |
| --- | --- | --- | --- | --- | --- |
| Age | 1.04 | 1.02 | 1.07 | <0.01 | 1.13 |
| F-type | 1.20 | 0.76 | 1.87 | 0.44 | 1.39 |
| CIS | 1.50 | 1.00 | 2.26 | 0.05 | 1.03 |
| CB | 2.18 | 1.32 | 3.62 | <0.01 | 1.03 |
| Sex | 1.15 | 0.77 | 1.74 | 0.49 | 1.09 |
| DM | 0.86 | 0.51 | 1.46 | 0.57 | 1.05 |
| S-time | 1.01 | 1.00 | 1.01 | 0.17 | 1.15 |
| HB | 1.00 | 0.99 | 1.01 | 0.64 | 1.35 |
| SII | 1.00 | 1.00 | 1.00 | 0.18 | 1.03 |

Supplementary Table 5. The composition of each model of NLR and the VIF values of all variables.

Supplementary Table 5-1. Model 1: No variables adjusted.

| Variable | OR | Lower-CI | Upper-CI | P-value |
| --- | --- | --- | --- | --- |
| NLR | 1.04 | 1.00 | 1.08 | 0.04 |

Supplementary Table 5-2. Model 2: Adjusted for age, F-type, CIS, and CB.

| Variable | OR | Lower-CI | Upper-CI | P-value | VIF |
| --- | --- | --- | --- | --- | --- |
| Age | 1.04 | 1.01 | 1.07 | <0.01 | 1.04 |
| F-type | 1.25 | 0.85 | 1.84 | 0.25 | 1.03 |
| CIS | 1.49 | 0.99 | 2.22 | 0.05 | 1.01 |
| CB | 2.24 | 1.36 | 3.68 | <0.01 | 1.01 |
| NLR | 1.03 | 0.99 | 1.07 | 0.17 | 1.02 |

Supplementary Table 5-3. Model 3: Adjusted for age, F-type, CIS, CB, sex, DM, S-time, and HB.

| Variable | OR | Lower-CI | Upper-CI | P-value | VIF |
| --- | --- | --- | --- | --- | --- |
| Age | 1.04 | 1.02 | 1.07 | <0.01 | 1.14 |
| F-type | 1.20 | 0.76 | 1.88 | 0.43 | 1.39 |
| CIS | 1.51 | 1.00 | 2.26 | 0.05 | 1.03 |
| CB | 2.19 | 1.32 | 3.63 | <0.01 | 1.03 |
| Sex | 1.14 | 0.76 | 1.72 | 0.52 | 1.09 |
| DM | 0.87 | 0.51 | 1.47 | 0.59 | 1.05 |
| S-time | 1.00 | 1.00 | 1.01 | 0.19 | 1.15 |
| HB | 1.00 | 0.99 | 1.01 | 0.62 | 1.35 |
| NLR | 1.02 | 0.98 | 1.07 | 0.23 | 1.04 |
